# Propolis Protects GC-1spg Spermatogonial Cells against Tert-Butyl Hydroperoxide-Induced Oxidative Damage

**DOI:** 10.3390/ijms25010614

**Published:** 2024-01-03

**Authors:** Filipa Duarte, Mariana Feijó, Ângelo Luís, Sílvia Socorro, Cláudio J. Maia, Sara Correia

**Affiliations:** CICS-UBI—Health Sciences Research Centre, University of Beira Interior, Av. Infante D. Henrique, 6200-506 Covilhã, Portugal; filipa.duarte@ubi.pt (F.D.); mariana@fcsaude.ubi.pt (M.F.); afluis27@gmail.com (Â.L.); ssocorro@fcsaude.ubi.pt (S.S.); cmaia@fcsaude.ubi.pt (C.J.M.)

**Keywords:** antioxidant, oxidative stress, propolis, spermatogonial cells, tert-butyl hydroperoxide

## Abstract

Propolis is a natural resin produced by honeybees with plenty of pharmacologic properties, including antioxidant activity. Oxidative stress disrupts germ cell development and sperm function, with demonstrated harmful effects on male reproduction. Several natural antioxidants have been shown to reduce oxidative damage and increase sperm fertility potential; however, little is known about the effects of propolis. This work evaluated the role of propolis in protecting spermatogonial cells from oxidative damage. Propolis’ phytochemical composition and antioxidant potential were determined, and mouse GC-1spg spermatogonial cells were treated with 0.1–500 µg/mL propolis (12–48 h) in the presence or absence of an oxidant stimulus (tert-butyl hydroperoxide, TBHP, 0.005–3.6 µg/mL, 12 h). Cytotoxicity was assessed by MTT assays and proliferation by Ki-67 immunocytochemistry. Apoptosis, reactive oxygen species (ROS), and antioxidant defenses were evaluated colorimetrically. Propolis presented high phenolic and flavonoid content and moderate antioxidant activity, increasing the viability of GC-1spg cells and counteracting TBHP’s effects on viability and proliferation. Additionally, propolis reduced ROS levels in GC-1spg, regardless of the presence of TBHP. Propolis decreased caspase-3 and increased glutathione peroxidase activity in TBHP-treated GC-1spg cells. The present study shows the protective action of propolis against oxidative damage in spermatogonia, opening the possibility of exploiting its benefits to male fertility.

## 1. Introduction

Propolis or bee glue is a natural substance synthesized by honeybees from numerous plant resinous secretions, such as blossoms, gums, and plants, and also from leaf buds of different plant species like pines, palms, alder, poplar, conifer, and then mixed with salivary and enzymatic secretions [1,2,3,4,5,6]. The area and the plant source can influence the color of propolis, as well as its composition [3]. Propolis chemical composition is not well-defined and differs considerably from region to region along with vegetation, from season to season, and from hive to hive. As a result of its great variability, more than 300 different chemical compounds have been identified in propolis [2,3,6,7]. Propolis typically includes 50% plant resins, 30% waxes, 10% essential and aromatic oils, 5% pollens, and 5% other organic substances. Nevertheless, the main chemical groups present in propolis resin comprise phenolic acids or their esters, flavonoids, terpenes, aromatic aldehydes and alcohols, fatty acids, stilbenes, and β-steroids [2,3,8]. Phenolic compounds are the main compounds of propolis and are responsible for its pharmacological activity [9]. Among them, flavonoids, flavones, and terpenoids could be found, contributing not only to the scent but also to the biological properties of this natural substance [1,2,9]. Nowadays, propolis is used as an antibacterial and antifungal, anti-inflammatory, antiviral, antioxidant, anesthetic, antitumoral, antiproliferative, antimutagenic, and antihepatotoxic agent, in addition to being used for cytotoxic activity and in neurodegenerative and depressive diseases [1,2,3]. It is found commercially in the form of dentifrices, mouth rinses, antiseptic mixtures, cough syrups, soap, creams, gels, shampoos, lotions, as well as candies, powder, cakes, wine, chewing gums, and chocolate bars [3,10,11,12,13]. To generally healthy humans, a safe dose of propolis has been reported to be 70 mg/day [9,14].

The antioxidant activity of propolis is due to its enrichment of polyphenols such as phenolic acids and flavonoids, with most outcomes demonstrating a reduction in oxidative stress (OS) markers [1,9,15]. These compounds of propolis may enter the skin and protect the epidermis and dermis from free radicals, which are produced due to radiations or premature aging of dermal cells [3]. To reduce OS-induced tissue damage, endogenous antioxidant systems have developed protective enzymatic and non-enzymatic mechanisms [9,14,16]. The chemical structure of polyphenols allows propolis to remove free radicals, which are the primary cause of lipids, nucleic acids, and protein oxidation, inhibiting the formation of reactive oxygen species (ROS), chelating metal ions, and exerting synergistic roles with other antioxidants [2,3,9,17]. The antioxidant activity of propolis has also been connected to its protective effect against heavy metal toxicity, cardiovascular anomalies, insufficient liver function, and brain pathologies [3,18]. Portuguese propolis is rich in phenols and has an important capacity for scavenging free radicals and preventing lipid peroxidation [1,3]. 

Many genetic, environmental, and physiological factors have been implicated in defective sperm function, the most common cause of male infertility [19,20]. Sperm damage by ROS has gained considerable attention for its role in inducing poor sperm function and infertility [19,20]. Products that can offer spermatozoa protection are, therefore, of great importance [19]. In this context, natural products, like garlic, ginger, grapes, and propolis, have been studied as protective agents and eventually as new supplementary therapeutic options for the treatment of male sexual disorders [19,21,22,23]. Specifically, propolis has been suggested as a protective factor against different OS inducers in the testis due to its ability to scavenge free radicals [1,21]. Administration of propolis in rats and rabbits shows an improvement in body and reproductive organs’ weight, an increase in sperm production, motility, count, and quality, and a reduction in abnormal and dead sperm numbers [19,22,23,24,25]. In co-administration with OS-inducers, like cadmium, aluminum, and copper, propolis ameliorated testicular histopathological and biochemical alterations, decreased the number of apoptotic cells, increased the levels of antioxidant enzymes, with a simultaneous decrease in malonaldehyde levels, a marker of lipid peroxidation [19,21,26,27]. In men, propolis administration was demonstrated to reduce the free radical-induced lipid peroxidation, as well as increase superoxide dismutase (SOD) activity [22]. Further, it also showed the capacity to protect spermatozoa’s DNA and membrane from oxidative damage [19,22]. Moreover, like in rats, in men, the reproductive organs’ weight is also restored, and there is an increase in sperm production, motility, and concentration, as well as a reduction in abnormal and dead sperm [28].

Despite all the evidence associating the propolis’ ability to reduce OS effects in male reproductive function, little is known about the effect of this natural product on specific testicular cell populations. Most studies focus on spermatozoa instead of upstream cells or events, as is the extremely coordinated and complex process of spermatogenesis, where oxidative damage may also be deleterious for male fertility. Attempting to fill this lack of knowledge, the present work aimed to analyze the preventive potentialities of propolis towards spermatogonial oxidative damage. For this purpose, a mouse-derived spermatogonial cell line (GC-1spg) was exposed to the OS inducer tert-butyl hydroperoxide (TBHP) in the presence or absence of propolis, assessing GC-1spg cell viability, apoptosis, proliferation, ROS levels, and antioxidant defenses.

## 2. Results

### 2.1. Propolis’ Characterization: Phytochemical Composition, Antioxidant Properties and Cytotoxicity

Phenolic compounds and flavonoids were reported to protect the reproductive system against toxic substances. Therefore, the content of phenolics and flavonoids from the propolis used in this research was analyzed. Total phenolics and flavonoids were in a proportion of 168.70 ± 0.08 mg gallic acid equivalents (GAE)/g propolis and 36.90 ± 0.19 mg quercetin equivalents (QE)/g propolis, respectively, which is equivalent to 16.87% total phenolics and 3.69% flavonoids (Table 1). 

The antioxidant activity of the studied propolis was also assessed to evaluate its potential to prevent or reduce OS. Propolis presented moderate antioxidant activity, as its antioxidant activity index (AAI) values were between 0.5 and 1.0, and the half maximal inhibitory concentration (IC_50_) was 63.32 ± 13.04 µg/mL (Table 1).

**Figure 1:**
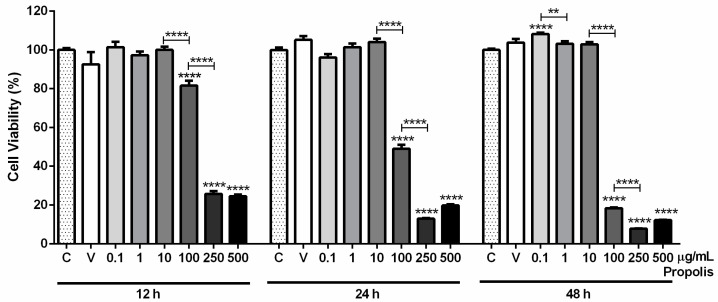
Viability of GC-1spg cells in the presence of different concentrations of propolis (0.1, 1, 10, 100, 250, and 500 µg/mL) for 12 h, 24 h, and 48 h, evaluated by MTT assay. Results are expressed as % of the control group (C). Ethanol in a concentration of 0.61% was used as a vehicle control (V). Data are presented as mean ± S.E.M. (6 replicates/group, mean of two independent assays). (*) Statistically significant differences to control (above error bars) and between consecutive concentrations (above bounding lines). ** *p* < 0.01; **** *p* < 0.0001.

The viability of GC-1spg cells after exposure to 0.1, 1, 10, 100, 250, or 500 µg/mL propolis for 12, 24, and 48 h was measured by the 3-(4,5-dimethylthiazol-2-yl)-2,5-diphenyltetrazolium bromide (MTT) assay. Ethanol 0.61%, which corresponds to the amount of solvent at the higher concentration of propolis, was used as a vehicle control. At this concentration, ethanol did not affect the viability of GC-1spg cells, as no differences were observed between vehicle and control groups at any exposure time (12 h: 100.00 ± 0.93% in control vs. 92.61 ± 6.21% in vehicle; 24 h: 100.00 ± 1.26% in control vs. 105.20 ± 2.05% in vehicle; 48 h: 100.00 ± 0.73% in control vs. 103.80 ± 1.88% in vehicle; Figure 1).

The results from 12 h showed no significant differences in cell viability at lower concentrations of propolis (control: 100.00 ± 0.93%; 0.1 µg/mL: 101.40 ± 2.82%; 1 µg/mL: 97.28 ± 1.86%; 10 µg/mL: 100.00 ± 1.65%; Figure 1). At higher concentrations, propolis caused a substantial decrease in the viability of GC-1spg cells (control: 100.00 ± 0.93% vs. 100 µg/mL: 81.56 ± 2.54%, *p* < 0.0001; 250 µg/mL: 25.69 ± 1.53%, *p* < 0.0001; and 500 µg/mL: 24.53 ± 0.79%, *p* < 0.0001; Figure 1). Between 10 and 100 µg/mL and 100 and 250 µg/mL, the viability decreased significantly (18.46%, *p* < 0.0001 and 55.87%, *p* < 0.0001, respectively).

After 24 h, the results were similar, with no impact in cell viability at lower concentrations (control: 100.00 ± 1.26%; 0.1 µg/mL: 96.15 ± 1.69%; 1 µg/mL: 101.50 ± 1.88%; 10 µg/mL: 104.10 ± 1.73%; Figure 1) and reduced GC-1spg viability when treated with 100, 250 and 500 µg/mL propolis (control: 100.00 ± 1.26% vs. 100 µg/mL: 49.06 ± 2.01%, *p* < 0.0001; 250 µg/mL: 12.98 ± 0.32%, *p* < 0.0001; and 500 µg/mL: 19.77 ± 0.62%, *p* < 0.0001; Figure 1). Between 10 and 100 µg/mL and 100 and 250 µg/mL, the viability also decreased significantly (55.02% and 36.09% less, respectively, both *p* < 0.0001).

When exposed to propolis for 48 h, a significant increase in the viability of GC-1spg cells was noticed at 0.1 µg/mL concentration (control: 100.00 ± 0.73% vs. 0.1 µg/mL: 108.20 ± 0.70%, *p* < 0.0001; Figure 1). The 1 and 10 µg/mL concentrations showed no significant differences (control: 100.00 ± 0.73%; 1 µg/mL: 103.20 ± 1.22%; 10 µg/mL: 103.00 ± 1.15%; Figure 1), whereas, at 100, 250 and 500 µg/mL, propolis caused a substantial decrease in cell viability (control: 100.00 ± 0.73% vs. 100 µg/mL: 18.34 ± 0.47%, *p* < 0.0001; 250 µg/mL: 7.84 ± 0.09%, *p* < 0.0001; and 500 µg/mL: 12.25 ± 0.13%, *p* < 0.0001; Figure 1). The viability of GC-1spg cells was significantly decreased between 0.1 and 1 µg/mL, 10 and 100 µg/mL, and 100 and 250 µg/mL (5.00% less, *p* < 0.01, 84.61% less, *p* < 0.0001, and 10.51% less, *p* < 0.001, respectively; Figure 1).

After analysis and integration of the obtained results, the 0.1 µg/mL and 1 µg/mL concentrations of propolis and the 24 h time point were selected for the subsequent analyses, as the viability of GC-1spg cells was not affected.

### 2.2. Propolis Attenuated the Impact of the OS Inducer Tert-Butyl Hydroperoxide (TBHP) in the Viability of GC-1spg Cells

As the main aim of this study was to demonstrate the antioxidant potential of propolis, TBHP was used as an OS inducer that has been widely associated with testicular oxidative damage. 

**Figure 2:**
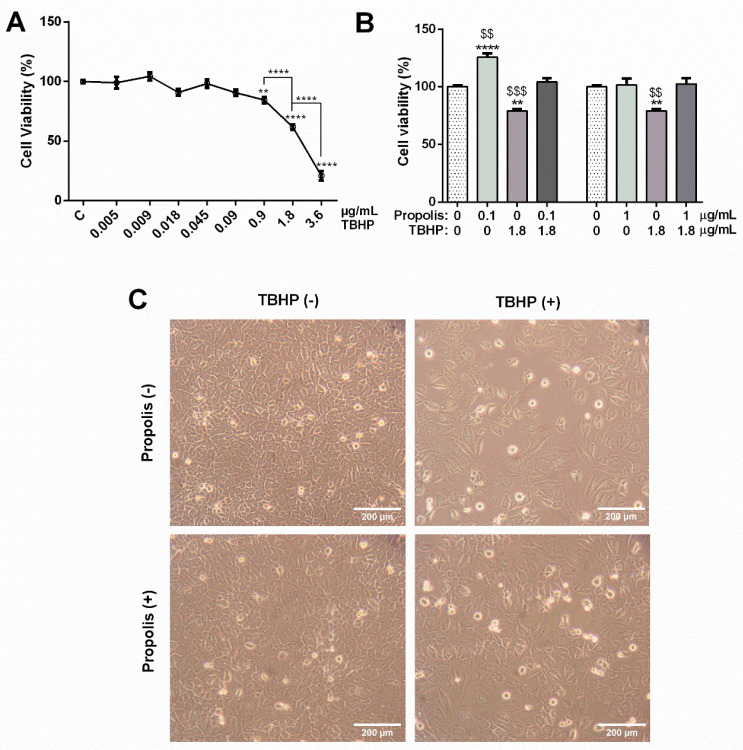
Propolis effect on tert-butyl hydroperoxide (TBHP) cytotoxicity in GC−1spg cells. (**A**) Viability of GC-1spg cells in the presence of different concentrations of TBHP (0.005, 0.009, 0.018, 0.045, 0.09, 0.9, 1.8 and 3.6 µg/mL) for 12 h. (**B**) Viability of GC-1spg cells in the presence of 0.1 or 1 µg/mL propolis alone for 36 h, and after a propolis’ 24 h-pre-treatment followed by 12 h exposure to 1.8 µg/mL TBHP (with or without propolis). Cell viability was evaluated by MTT assay. Results are expressed as % of control group. Data are presented as mean ± S.E.M. (6 replicates/group, mean of two independent assays). (*) Statistically significant difference when compared to control; (^$^) Statistically significant difference when compared to TBHP plus propolis respective group. ** *p* < 0.01; **** *p* < 0.0001; ^$$^ *p* < 0.01; ^$$$^ *p* < 0.001. (**C**) Representative images of GC-1spg cells from each study condition (control, 1.8 µg/mL TBHP, 0.1 µg/mL propolis, and both). Micrographs were taken by Olympus CKX41, with an ampliation of 100×. The scale bar represents 200 µm.

The viability of GC-1spg cells after 12 h of exposure to different concentrations of TBHP (0.005, 0.009, 0.018, 0.045, 0.09, 0.9, 1.8 and 3.6 µg/mL) was evaluated by MTT assay. Concentrations in the range of 0.005 to 0.09 µg/mL showed no significant differences on cell viability when compared to control (control: 100.00 ± 1.43%; 0.005 µg/mL: 98.99 ± 4.79%; 0.009 µg/mL: 104.30 ± 3.32%; 0.018 µg/mL: 90.87 ± 2.83%; 0.045 µg/mL: 98.19 ± 3.47%; 0.09 µg/mL: 90.61 ± 2.70%; Figure 2A). Concentrations above 0.09 µg/mL TBHP reduced the viability of GC-1spg cells (control: 100.00 ± 1.43% vs. 0.9 µg/mL: 84.41 ± 2.64%, *p* < 0.01; 1.8 µg/mL: 61.71 ± 2.22%, *p* < 0.0001; and 3.6 µg/mL: 20.77 ± 3.88%, *p* < 0.0001; Figure 2A). The effect was concentration-dependent, as significant differences were observed between 0.9 and 1.8 µg/mL TBHP (22.71% decrease, *p* < 0.0001; Figure 2A) and between 1.8 and 3.6 µg/mL TBHP (40.94% decrease, *p* < 0.0001; Figure 2A).

The 1.8 µg/mL concentration of TBHP was selected for the subsequent analyses, as the accentuated decrease in cell viability after exposure to 3.6 µg/mL TBHP (only ≈21% viable cells) may difficult the study of the molecular mechanisms underlying GC-1spg cell damage induced by TBHP. To study the protective potential of propolis against TBHP cytotoxicity in GC-1spg cells, cells were exposed to 1.8 µg/mL TBHP after 24 h-pre-treatment with propolis (0.1 and 1 µg/mL). Despite the fact that 1 µg/mL propolis had no effect (control: 100.00 ± 2.06%; 1 µg/mL propolis: 101.70 ± 5.62%; Figure 2B), the lowest concentration increased the viability of GC-1spg cells (control: 100.00 ± 2.06% vs. 0.1 µg/mL propolis: 125.70 ± 3.55%, *p* < 0.0001; Figure 2B). After exposure to 1.8 µg/mL TBHP, GC-1spg viability was significantly decreased (control: 100.00 ± 2.06% vs. 1.8 µg/mL TBHP: 79.04 ± 1.76%, *p* < 0.01; Figure 2B). Propolis attenuated the TBHP effect, restoring cell viability to that of control levels, at any of the selected concentrations (without propolis: 79.04 ± 1.76% vs. 0.1 µg/mL propolis: 104.20 ± 3.18%, *p* < 0.001; and 1 µg/mL propolis: 102.50 ± 5.02%, *p* < 0.01; Figure 2B). 

Considering the results, the lowest concentration of propolis (0.1 µg/mL) was selected for the subsequent analyses, as both propolis concentrations reversed the TBHP effect, and 0.1 µg/mL propolis was also able to increase cell viability in the absence of TBHP. Representative micrographs of GC-1spg cells from each study condition (control, 1.8 µg/mL TBHP, 0.1 µg/mL propolis, and both) are shown in Figure 2C.

### 2.3. Propolis Prevented the Antiproliferative Effect of TBHP in GC-1spg Cells, Reducing Caspase-3 Activity Only in the Presence of TBHP Stimuli

**Figure 3:**
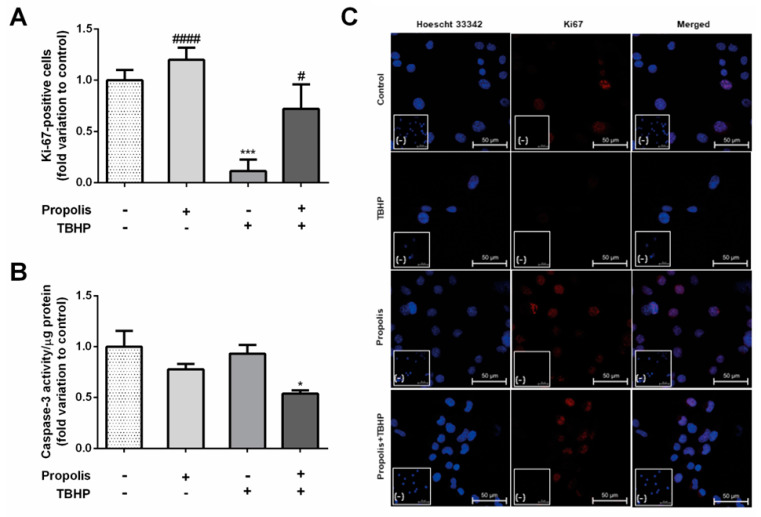
Proliferative status (**A**) and caspase-3 activity (**B**) of GC−1spg cells cultured for 24 h in the presence of 0.1 µg/mL propolis followed by 12 h in the presence of 1.8 µg/mL tert-butyl hydroperoxide (TBHP, with or without propolis). Proliferative index was determined by counting the number of K-i67-positive cells normalized with Hoechst-stained cells. Results are expressed as fold variation to the control group. Data are presented as mean ± S.E.M. (triplicates/group, two independent assays). (*) Statistically significant difference when compared to control; (^#^) Statistically significant difference when compared to TBHP group. * *p* < 0.05, *** *p* < 0.001; ^#^ *p* < 0.05; ^####^ *p* < 0.0001. (**C**) Representative confocal microscopy images showing Ki-67 labeling in the different groups. Images were taken by Zeiss LMS 710 laser scanning confocal microscope under 400× magnification. The scale bar represents 50 µm. Ki-67 positive cells are shown as red, and the nuclei are stained blue (Hoechst 33342). Negative controls were obtained by omission of the primary antibody and provided as insert panels (-).

Cell proliferation index was determined by the number of Ki-67-positive cells relative to the total cell number, as this protein is detected in the nucleus of proliferating cells in all active phases of the cell division cycle. TBHP-treated cells showed a significantly decreased proliferation index when compared to control (TBHP: 0.11 ± 0.11–fold variation to control, *p* < 0.001; Figure 3A). In the presence of propolis, there were no significant differences in the number of Ki-67-positive cells (propolis: 1.20 ± 0.11–fold variation to control; TBHP + propolis: 0.72 ± 0.24–fold variation to control; Figure 3A).

Caspase-3 has been recognized as an endpoint of apoptosis, activated in apoptotic cells by both extrinsic (death ligands) and intrinsic (mitochondrial) pathways. Here, neither propolis nor TBHP altered caspase-3 activity (propolis: 0.78 ± 0.05-fold variation to control; TBHP: 0.93 ± 0.09-fold variation to control; Figure 3B). However, propolis significantly decreased caspase-3 activity when cells were exposed to TBHP (TBHP + propolis: 0.54 ± 0.03–fold variation to control, *p* < 0.05; Figure 3B).

### 2.4. Propolis Reduced Reactive Oxygen Species (ROS) Levels in GC-1spg Cells, Raising Glutathione Peroxidase (GPx) Activity in Cells Exposed to TBHP

**Figure 4:**
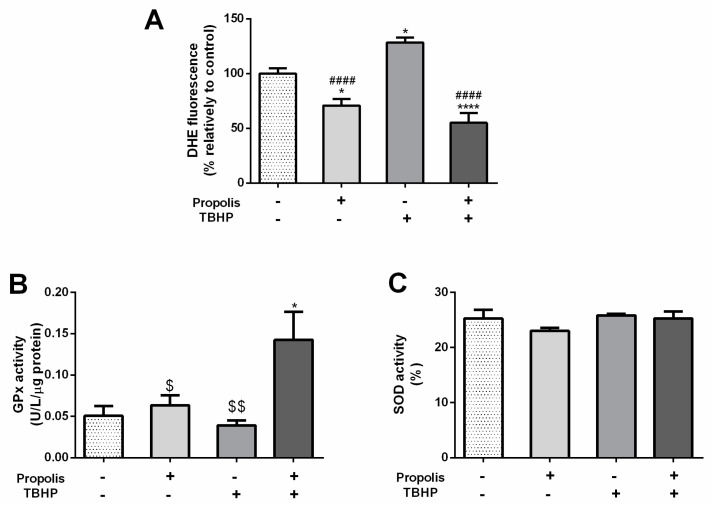
ROS levels (**A**), glutathione peroxidase (GPx, **B**), and superoxide dismutase (SOD, **C**) activity in GC-1spg cells cultured for 24 h in the presence of 0.1 µg/mL propolis followed by 12 h in the presence of 1.8 µg/mL tert-butyl hydroperoxide (TBHP, with or without propolis). Dihydroethidium (DHE) fluorescence normalized with Hoechst, and results are expressed as % of the control group. Data are presented as mean ± S.E.M. (triplicates/group, two independent assays). (*) Statistically significant difference when compared to control; (^#^) Statistically significant difference when compared to TBHP group. (^$^) Statistically significant difference when compared to TBHP plus propolis group. * *p* < 0.05; **** *p* < 0.0001; ^####^ *p* < 0.0001; ^$^ *p* < 0.05; ^$$^ *p* < 0.01.

Dihydroethidium (DHE) acts as a fluorescent probe for the detection of ROS generation and is specific for superoxide and hydrogen peroxide. DHE results were normalized with Hoechst fluorescence and expressed as % relative to the control group. TBHP-treated cells showed a significant increase in DHE fluorescence when compared to the control group (control: 100.00 ± 4.78% vs. TBHP: 128.30 ± 4.54%, *p* < 0.05; Figure 4A). Noteworthy, the intensity of fluorescence was significantly decreased in the propolis study conditions, regardless of TBHP exposure (control: 100.00 ± 4.78% vs. propolis: 70.85 ± 5.99%, *p* < 0.05; and TBHP + propolis: 55.04 ± 8.97%, *p* < 0.0001 Figure 4A). This resulted in a decrease in DHE fluorescence of 73.24%, *p* < 0.0001 when comparing TBHP exposure with or without propolis.

Glutathione peroxidase (GPx) is an important intracellular enzyme that breaks down H_2_O_2_ to H_2_O. In the presence of TBHP, it was observed a decrease in the activity of GPx, despite not being significantly different when compared to the control group (control: 0.05 ± 0.01 U/L/µg protein; TBHP: 0.04 ± 0.01 U/L/µg protein; Figure 4B). The activity of GPx was only significantly increased when GC-1spg cells were treated with propolis and TBHP (control: 0.05 ± 0.01 U/L/µg protein vs. TBHP + propolis: 0.14 ± 0.03 U/L/µg protein, *p* < 0.05; propolis: 0.06 ± 0.01 U/L/µg protein; Figure 4B), being significantly increased when compared to TBHP or propolis groups (increase of 0.10 U/L/µg protein, *p* < 0.01 and 0.08 U/L/µg protein, *p* < 0.05, respectively; Figure 4B).

SOD catalyzes the dismutation of O_2_^·−^ into molecular O_2_ and H_2_O_2_, consequently rendering the potentially harmful superoxide anion less hazardous. Herein, no significant differences in any experimental group were observed concerning SOD activity (control: 25.28 ± 1.55%; TBHP: 25.85 ± 0.28%; propolis: 23.03 ± 0.54%; TBHP + propolis: 25.28 ± 1.25%; Figure 4C).

## 3. Discussion

In the present work, the ability of propolis to prevent oxidative damage induced by TBHP in spermatogonial cells was studied. Firstly, propolis’ phytochemical composition and antioxidant properties were evaluated. Here, an aim-focused characterization was performed, as it is already commercialized. However, especially in the case of crude extracts, the panoply of propolis’ potentialities (antioxidant, anti-inflammatory, antibacterial, antifungal, antiviral, anesthetic, antimutagenic), as well as the influence of geographic and climatic conditions in its constituents, require a deeper analysis with a full characterization (e.g., liquid chromatography-mass spectrometry) to better explore the overall benefits of this resin. The propolis here studied presented a content of total phenolic and flavonoid of 168.7 ± 0.08 mg GAE/g and 36.9 ± 0.08 mg QE/g, being in the same range as in areas considered rich in these compounds, such as Spain and Turkey, where total phenolics range from 200 to 340 and 115 to 210 mg GAE/g, respectively, and the flavonoid content from 72–161 mg QE/g in Spain to 13–379 mg QE/g in Turkey. In the south hemisphere, the values decrease to 127–142 mg GAE/g and 33–53 mg QE/g in Brazil [29]. Noteworthy, our values are also in the same range as the ones from Chinese (135.26 mg GAE/g and 29.03 mg QE/g) and Egyptian (200.70 mg GAE/g and 91.86 mg QE/g) propolis used in a recent study demonstrating their preventive potentialities in male reproductive function impairment [27]. The chemical composition of propolis is strongly dependent on the plant sources available around the hive, the geographical and climatic conditions, as well as the surrounding flora [30,31,32]. Propolis from Europe and North America show similar phenolic composition, with the main compounds being flavonoids, phenolic acids, and their esters [30]. Moreover, terpenes were the major components in propolis from Mediterranean Sea areas and tropical areas, particularly in Brazil, where propolis presented a composition rich in prenylated phenylpropanoids and caffeoylquinic acid [30]. Flavonoids, as one of the most diverse and widespread groups of natural compounds, are probably the most important phenolic compounds [33]. These compounds possess a broad spectrum of biological and chemical activities, including radical scavenging properties [33]. The IC_50_ value, obtained via the DPPH assay, was 63.32 ± 13.04 µg/mL, and as the value is inversely proportional to the antioxidant activity, the results demonstrated a moderate antioxidant activity with an AAI of 0.77 ± 0.13. Indeed, the antioxidant activity of propolis is basically due to the presence of phenolic compounds and flavonoids, although the exact mechanism of action is unknown [33,34]. Among the mechanisms proposed are free radical scavenging, hydrogen donation, metallic ion chelation, or the capability to act as a substrate for radicals such as superoxide and hydroxyl [34]. As mentioned, propolis is known to contain large amounts of polyphenols and flavonoids, which are strongly related to its ROS-scavenging properties [35]. In general, DPPH free radical-scavenging activity ranged from about 20 to 190 μg/mL [36,37]. The free radical scavenging activity analyzed in this work is within this range once the results showed an IC_50_ of 63.32 ± 13.04 µg/mL. In sum, the present results demonstrated that its phytochemical properties and antioxidant potential are similar to the ones found in the literature.

To evaluate the potential protective role of propolis in cells, the viability of GC-1spg cells treated with propolis was analyzed. In the three time points chosen (12, 24, and 48 h), at higher concentrations (100, 250, and 500 µg/mL), propolis caused a substantial decrease in cell viability. At low concentrations (0.1 and 1 µg/mL, 24 h), the viability of GC-1spg was not affected. When exposed to the well-known OS inducer TBHP, the viability of GC-1spg cells decreased in a concentration-dependent manner, and the concentration of 1.8 µg/mL TBHP (equivalent to 20 µM) was selected. TBHP cytotoxic assays in murine hepatocytes demonstrated a 40%-cell viability reduction at a concentration of 250 µM and a total loss of viability with 500 µM TBHP [38,39]. In the human normal liver cell line QZG, the viability decreased markedly in cells treated with 200 µM–8 mM TBHP, and concentrations higher than 64 µM TBHP reduced human prostate epithelial cells viability by 98% [40,41]. In general, the concentration range of TBHP in the above-mentioned studies was higher than that used in the present work. Indeed, this observation enforces the sensitivity of GC-1spg to noxious stimuli and, in particular, to the disruptive effects of TBHP, prompting the emergence of preventive approaches. 

When GC-1spg cells were treated with propolis and TBHP, propolis attenuated the TBHP effect. Indeed, propolis has been reported to control cell growth and viability, presenting selectivity between normal and cancer cell lines due to its different types of compounds [42]. On the one hand, propolis has an inhibitory effect against several cancer cells via an increase in apoptosis, and on the other hand, the flavonoids present in this resin are powerful antioxidants and capable of scavenging free radicals and thereby protecting normal cells [42]. In fact, previous studies in Caco-2 cells (a cell line from colon carcinoma) showed that 1 mg/mL propolis decreased cell viability by around 25% [43,44], while in normal cells, such as human lymphocytes, rat kidneys, liver, and spleen, the same concentration stimulated cell growth [42]. In the present work, a concentration as low as 0.1 µg/mL propolis preserved GC-1spg cell viability upon the noxious stimuli of TBHP. Corroborating our results, the preventive potentialities of propolis have been studied against other damaging factors, such as anticancer drugs [28]. A study with male rats demonstrated that the sperm viability after a 4-week treatment with the anticancer drug paclitaxel decreased to 23%, and when in the presence of propolis, this viability was re-established to 69% [28]. Similar results were observed in rats, where propolis minimized the toxicity of chlorpyrifos in sperm [45]. 

The proper regulation of the caspase cascade plays an important role in male fertility, sperm differentiation, and testicular maturity [46,47]. Hence, the effect of propolis on the apoptosis of GC-1spg cells was evaluated by analyzing the activity of the endpoint regulator of the apoptotic pathway, caspase-3 [48]. Propolis alone did not affect the activity of caspase-3 on GC-1spg cells, but when in the presence of TBHP, the activity of caspase-3 was significantly decreased. Once again, the propolis effect was only observed in the presence of noxious stimuli. TBHP itself did not show any effect on caspase-3 activity, demonstrating that the reduction in GC-1spg cell viability by TBHP is not attributed to programmed cell death. In previous studies, propolis was able to attenuate the effect of damaging stimuli-induced apoptosis in germ cells, as well as in rat sperm [25,49,50]. When exposed to cadmium, the number of apoptotic cells in testicular tissue increased in rats, but when pre-treated with propolis, the number of apoptotic cells was similar to that of the control group [25]. Similar results were obtained in rats exposed to doxorubicin and in mice exposed to mitomycin C [49,50]. Khayyal et al. attributed this anti-apoptotic effect of propolis to its content of aromatic acids, including phenolic and caffeic acids [51]. In the present work, a significant decrease in the proliferation of GC-1spg cells when exposed to the OS inducer TBHP was observed. During the normal spermatogenic cycle, there is active proliferation of A_s_, A_pr,_ and A_al_ spermatogonia in mice and little or no proliferation in the remaining stages [52]. However, when the numbers of A4, Intermediate, and B spermatogonia are low, the proliferation period is extended by a feedback mechanism. This way, any stimuli affecting spermatogonia proliferative capability could be detrimental to the spermatogenic process. Again, the protective role of propolis was demonstrated as it re-established the proliferative capability of GC-1spg cells. This observation is in line with the study from Najafi et al., where, after being exposed to propolis, human lymphocytes, rat kidney, liver and rat spleen cells showed a faster rate of cell proliferation and increased cell number [42]. 

ROS are normally produced during metabolic events of living cells, particularly by the mitochondrial electron-transport chain [24,53]. However, high levels of ROS result from the dysregulated balance between their production and elimination, inducing OS, as well as many diseases such as cardiovascular diseases, diabetes mellitus, and cancer [53,54]. As expected, after exposure to the well-known OS inducer TBHP, ROS significantly increased in GC-1spg cells. This overproduction of ROS following TBHP exposure agrees with several studies on mice testis and sperm [54,55,56,57]. Relatively to propolis, its antioxidant potential was corroborated as it caused a significant decrease in GC-1spg ROS levels. According to the literature, the main antioxidant mechanisms of propolis are associated with the capacity of polyphenols to scavenge ROS and decrease xanthine oxidase activity, thus interrupting the cascade of reactions leading to the peroxidation of lipids [58,59,60]. These findings were also demonstrated by studies made in diabetic rats, human keratinocyte cells, and sperm, where they empathized that the antioxidant capacity of propolis was due to its phenolic compounds [61,62,63]. Phenolic acids are compounds built of a benzene ring, carboxyl, and hydroxyl groups, and their antioxidative activity depends on the number of hydroxyl groups in their molecules and on their steric effects [58]. Thereby, the position of hydroxyl groups, as well as the type of substitution on the aromatic ring, influence the antioxidative activity of these compounds [58].

To neutralize OS effects, testis exhibits antioxidant defense mechanisms that include antioxidant enzymes such as SOD and GPx [64,65]. SOD degrades superoxide into oxygen and hydrogen peroxide, with the last being further detoxified by GPx [66]. In this work, propolis increased GPx activity in GC-1spg cells, regardless of TBHP exposure. However, GPx levels appeared to be unaffected by propolis or TBHP alone, and neither propolis nor TBHP altered SOD activity. The main biological role of the GPx enzyme is to protect the organism from oxidative damage by catalyzing the conversion of H_2_O_2_ to H_2_O [54]. Despite constant GPx activity, ROS levels were elevated by TBHP treatment, raising the possibility that the detoxification of H_2_O_2_ was not being catalyzed as demanded, resulting in increased ROS that leads to OS [54]. In the literature, studies are controversial; once, on the one hand, TBHP induces a decrease in SOD and GPx activity in mice hepatocytes and, on the other hand, causes an increase in GPx level in mice testis [54,56,67]. Previous studies demonstrated that TBHP-treated rats presented a significant decrease in SOD activity in the testis while other studies in hepatocytes demonstrated an increase in its activity [55]. The discrepancies may be due to different exposure conditions, as well as to tissue/cell-specific features. Here, propolis only increased GPx activity in the presence of TBHP. In other studies performed in mice erythrocytes and rat testis, propolis increased GPx activity only at the highest dose, and the authors suggested that it was likely due to the pro-oxidant effect of that concentration of propolis [68,69]. These results led to the hypothesis that besides the dependence on the type of cell/tissue studied, the disparities in GPx activity can be concentration/dose-dependent. In other words, despite the fact that the low concentration of propolis alone did not affect GPx activity, propolis in higher concentration could by itself raise GPx activity, even in the absence of damaging stimuli. Some studies evidence a significant increase in SOD activity after propolis treatment in erythrocytes, testis, and sperm [14,21,26]. However, the results obtained here did not show any alterations in this detoxicant enzyme. According to a study in rats, the SOD defense system is only developed in late-stage spermatocytes at 4 weeks after birth [70], which may be the explanation for why SOD did not affect GC-1spg cells once they correspond to a spermatogonia B stage. 

The present work highlighted the benefits of natural compounds to male fertility, more precisely, the preventive potential of propolis against TBHP-induced OS in spermatogonial cells (GC-1spg). Indeed, the disruptive actions of TBHP were counteracted or even neutralized in the presence of propolis. Although preliminary, these findings highlight propolis role as a protective agent against OS, which is crucial in the context of spermatogenesis and male (in)fertility. 

## 4. Materials and Methods

### 4.1. Propolis and TBHP

Propolis (Biprol^®^, Myroxylon peruiferum, and Matriacaria chamomilla) was purchased in the form of a propolis ethanolic extract in a local store of natural products, and TBHP solution from Sigma-Aldrich (458139, CAS number: 75-91-2, St. Louis, MI, USA).

### 4.2. Total Phenolic Compounds Determination

The total phenolic compounds in propolis were determined by Folin–Ciocalteu’s colorimetric method [71]. Propolis was first diluted in methanol while gallic acid (used as standard) was diluted in water, and both were separately mixed with 0.2 N Folin–Ciocalteu’s reagent. The mixture was then incubated for 5 min, followed by the addition of aqueous Na_2_CO_3_ (75 g/L). After a 90-min incubation at 30 °C, the total phenols were determined spectrophotometrically at 765 nm (Helios–Omega, Thermo Scientific, Waltham, MA, USA). The standard curve was constructed using standard solutions of gallic acid in methanol, and the total phenolic values were expressed as mg GAE/g of extract.

### 4.3. Flavonoid Content Assessment

Propolis’ flavonoid content was determined by the aluminum chloride colorimetric method [71]. After dilution in methanol, propolis was mixed with 10% aluminum chloride and 1 M potassium acetate. This solution was incubated at room temperature for 30 min, and the absorbance of the reaction mixture was measured at 415 nm (Helios–Omega, Thermo Scientific). The calibration curve was constructed by preparing quercetin solutions in methanol. Total flavonoid values were expressed as mg QE/g of extract. 

### 4.4. 2,2-Diphenyl-1-picrylhydrazyl (DPPH) Free Radical Scavenging Assay

The antioxidant activity of propolis was determined by the radical scavenging activity method using the DPPH. Aliquots of methanolic solutions of the propolis at different concentrations were added to three DPPH methanolic solutions. After a 90-min incubation at room temperature in the dark, the absorbance was measured at 517 nm (Helios–Omega, Thermo Scientific). The radical scavenging activity was calculated by I% = [(Abs_0_ − Abs_1_)/Abs_0_] × 100. The IC_50_ was calculated using a calibration curve, and the AAI was calculated by AAI = final concentration of DPPH in the control sample/IC_50_ [71]. 

### 4.5. Cell Culture

A mouse-derived spermatogonial cell line (GC-1spg) corresponding to a spermatogonia type B stage (ATCC, CRL-2053™) was used in this work. 

GC-1spg cells were cultured and maintained in Dulbecco’s Modified Eagle Medium (DMEM, D0822, Sigma-Aldrich, USA), supplemented with 10% fetal bovine serum (FBS, P211107, PAN-Biotech, Aidenbach, Germany) and 1% penicillin-streptomycin-amphotericin B solution (A5955, Sigma-Aldrich), at 37 °C in an atmosphere of 5% CO2. The culture medium was replaced every 2–3 days, and cells were sub-cultivated each time they reached a confluence of 80–90%. When performing assays, the culture medium was replaced by phenol red-free DMEM (D1145, Sigma-Aldrich) containing 10% FBS (P211107, PAN-Biotech) and adjusting the concentration of L-glutamine (3.97 mM BP379, Thermo Fisher, USA), not present in this formulation.

### 4.6. Propolis and TBHP Treatments

For cell viability assays and ROS levels determination, approximately 2.5 × 10^3^ cells/well were grown in a 96-well plate (734-0023, VWR International, Radnor, PA, USA) and, in the remaining assays, approximately 2 × 10^5^ cells were seeded in each 25 cm^3^ T-flasks (70025, SPL Life Sciences, Pocheon-si, Gyeonggi-do, Korea).

**Figure 5:**
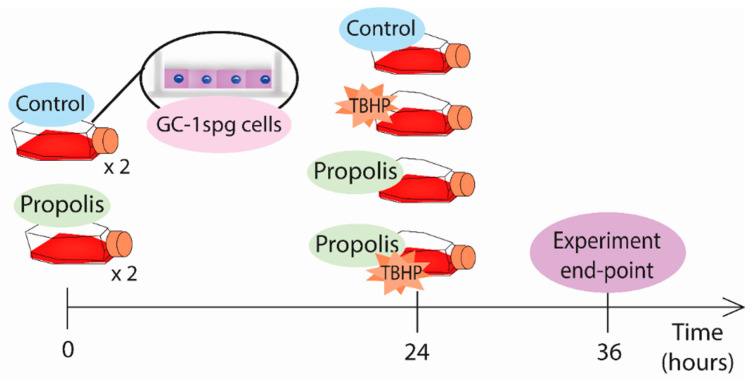
Experimental layout for propolis and tert-butyl hydroperoxide (TBHP) treatments. GC-1spg cells were first exposed to propolis for 24 h, followed by a 12 h exposure to either TBHP alone or propolis and TBHP.

To assess the cytotoxicity of TBHP, GC-1spg cells were exposed for 12 h to different concentrations (0.005, 0.009, 0.018, 0.045, 0.09, 0.9, 1.8, and 3.6 µg/mL) of this OS inducer. In the case of propolis, cells were treated during 12, 24, and 48 h to a 0.1, 1, 10, 100, 250, or 500 µg/mL concentration. When simultaneously assaying propolis and TBHP, cells were first exposed to propolis (0.1 and 1 µg/mL) for 24 h, followed by a 12 h exposure to either 1.8 µg/mL TBHP alone or propolis plus TBHP (Figure 5). Ethanol (0.61%) was used as a vehicle, corresponding to the percentage of ethanol in the most concentrated solution of propolis. The concentrations of 0.1 µg/mL propolis and 1.8 µg/mL TBHP were chosen to perform the subsequent analyses. 

### 4.7. 3-(4,5-Dimethylthiazol-2-yl)-2,5-diphenyltetrazolium Bromide (MTT) Assay

The viability of GC-1spg cells was evaluated by the MTT assay. After the aforementioned treatments, MTT (1 mg/mL, ab146345, Abcam, Cambridge, United Kingdom) was added to cells and incubated in the dark for 3 h at 37 °C. Then, the medium and MTT solution were carefully removed, and dimethyl sulfoxide (DMSO, 276855, Sigma-Aldrich) was added to dissolve the formazan crystals. The formazan content was measured at 570 nm using the xMark™ Microplate Absorbance Spectrophotometer (Bio-Rad Laboratories, Hercules, CA, USA). The value of absorbance was directly proportional to the number of viable cells. 

### 4.8. Ki-67 Fluorescent Immunohistochemistry

GC-1spg cells were fixed with 4% paraformaldehyde for 10 min and permeabilized with 1% Triton X-100 for 5 min at room temperature. Unspecific staining was blocked by incubation with phosphate-buffered saline (PBS) containing 0.1% (*w*/*v*) Tween-20 (PBST) and 20% FBS for 1 h. Then, cells were incubated with rabbit anti-Ki-67 (1:50, ab16667; Abcam) primary antibody for 1 h at room temperature. Alexa Fluor 546 goat anti-rabbit IgG was used as the secondary antibody (1:1000, 1813035; Invitrogen, Waltham, MA, USA). The specificity of the staining was assessed by the omission of primary antibody. Cell nuclei were stained with Hoechst 33342 (5 µg/mL, 1910197; Thermo Fisher) for 10 min. Lamellae were washed with PBST and mounted with Dako fluorescent mounting medium. Images were acquired using the Axio Imager A1 microscope (Carl Zeiss, Jena, Germany) and assessed with AxioVision 4.8 software (Carl Zeiss). 

### 4.9. Dihydroethidium (DHE) Assay

ROS levels were measured using the probe DHE as described by Georgiou et al. [33]. At the end of the exposure time, cells were cultured with 0.5 µM DHE for 20 min at 37 °C. After washing cells twice with PBS, the emitted fluorescence was read in a spectrofluorometer (SpectraMax M5e; Molecular Devices, San Jose, CA, USA) at 510 nm (excitation)/580 nm (emission). For cell number normalization, cell nuclei were stained with Hoechst 33342, and fluorescence read at 352 nm (excitation)/454 nm (emission).

### 4.10. Total Protein Extraction and Quantification

Total proteins were isolated from control, propolis, TBHP and propolis + TBHP-treated GC-1spg cells, using RIPA buffer (150 mM NaCl, 1% Nonidet-P40 substitute, 0.5% Na-deoxycholate, 0.1% SDS, 50 mM Tris pH 8.0 and 1 mM EDTA) supplemented with 1% protease-inhibitors cocktail (A7779, PanReac AppliChem, Darmstadt, Germany) and 10% phosphatase-inhibitors cocktail (4906845001, Roche, Basel, Switzerland). After being kept on ice for 30 min and homogenized, cells were centrifuged at 14,000 rpm, 20 min, 4 °C in a Hettich Mikro 200R centrifuge, and the supernatant containing proteins was collected. Total protein concentration was determined using the bicinchoninic acid method, using bovine serum albumin as standard. The proteins were stored at −80 °C.

### 4.11. Evaluation of Antioxidant Enzyme Activity

To analyze the antioxidant status of GC-1spg cells, the activity of the antioxidant enzymes GPx and SOD was evaluated.

GPx activity was determined using a commercial kit (703102, Cayman Chemical, Ann Arbor, MI, USA) according to the manufacturer’s protocol. GPx activity was measured by indirectly monitoring the glutathione reductase coupled reaction. Oxidized glutathione, produced in the reduction in an organic hydroperoxide by GPx, is recycled to its reduced state by glutathione reductase and NADPH. The oxidation of NADPH to NADP^+^ is accompanied by a decrease in absorbance at 340 nm [72]. Total protein extracts were incubated with NADPH, co-substrate, and GPx assay buffer. In the blanks, the sample was replaced using GPx assay buffer. The absorbance was measured at 340 nm every minute, and 7-time points were registered using the xMark™ Microplate Spectrophotometer (37 °C incubation, Bio-Rad). Under these conditions in which the GPx activity is rate limiting, the rate of decrease in absorbance is directly proportional to GPx activity in the sample. Results were expressed as U/L/µg of protein.

SOD activity was measured using a competitive inhibition assay (19160, Sigma-Aldrich) using a tetrazolium salt and xanthine oxidase, following the manufacturer’s instructions. Upon reduction with superoxide anion, a water-soluble formazan dye is produced, which is linear with xanthine oxidase activity and inhibited by SOD [73]. Briefly, total protein extracts were incubated with tetrazolium salt and xanthine oxidase (sample) or dilution buffer (sample blank). Two additional blanks were performed, the first with H_2_O, tetrazolium salt, and xanthine oxidase and the second with dilution buffer instead of the latter. The assay was monitored by measuring the absorbance at 450 nm, after a reaction time of 60 min at 37 °C, using the xMark™ Microplate Spectrophotometer (Bio-Rad). The percentage of reaction inhibition, extrapolated using the decrease in absorbance values, indicated the SOD activity. Results were expressed as the activity ratio (percentage of inhibition).

### 4.12. Caspase-3-like Activity Assay

Total protein extracts were incubated with assay buffer (20 mM HEPES pH 7.4; 2 mM EDTA; 0.1% 3-((3-cholamidopropyl) dimethylammonio)-1-propanesulfonate, CHAPS; 5 mM dithiothreitol DTT) and with 2 mM of caspase-3 substrate (AcDEVD-pNA; Sigma-Aldrich). Blanks were performed without protein. The incubation was undertaken overnight at 37 °C, and the absorbance values were read at 405 nm using the xMark™ Microplate Spectrophotometer (Bio-Rad). Upon caspase cleavage, pNA is released, producing a yellow color, which is measured spectrophotometrically at 405 nm. The amount of generated pNA was obtained by extrapolation with a standard curve of free pNA at different concentrations. Results were calculated as caspase-3 activity/µg protein and expressed as fold variation to control.

### 4.13. Statistical Analysis

Statistical analysis of all the obtained data was performed with the GraphPad Prism v6.01 software (GraphPad Software, San Diego, CA, USA). Statistically significant differences between the tested groups were obtained using one-way analysis of variance (ANOVA), followed by Tukey’s test. The differences were considered significant when *p* < 0.05 (* *p* < 0.05, ** *p* < 0.01, *** *p* < 0.001 and **** *p* < 0.0001). Experimental data are shown as mean ± standard error of the mean (S.E.M).

## Figures and Tables

**Table 1:** Phytochemical composition and antioxidant properties of propolis. Data are presented as mean ± S.E.M.

Total phenolics (mg GAE/g Propolis)	Flavonoids (mg QE/g Propolis)	IC_50_ (µg/mL)	Antioxidant Activity Index	Antioxidant Activity Classification
168.70 ± 0.08	36.90 ± 0.19	63.32 ± 13.04	0.77 ± 0.13	Moderate

## Data Availability

All the data are available in the present article.
